# Landsat phenological metrics and their relation to aboveground carbon in the Brazilian Savanna

**DOI:** 10.1186/s13021-018-0097-1

**Published:** 2018-05-15

**Authors:** M. Schwieder, P. J. Leitão, J. R. R. Pinto, A. M. C. Teixeira, F. Pedroni, M. Sanchez, M. M. Bustamante, P. Hostert

**Affiliations:** 1 Geography Department, Humboldt-Universität zu Berlin, Unter den Linden 6, 10099 Berlin, Germany; 2 Department Landscape Ecology and Environmental System Analysis, Institute of Geoecology, Technische Universität Braunschweig, Langer Kamp 19c, 38106 Braunschweig, Germany; 3 Departamento de Engenharia Florestal, Universidade de Brasília, Brasília, DF 70919-970 Brazil; 4 Graduate Program in Botany, University of Brasília, Brasília, DF 70919-970 Brazil; 5 Instituto de Ciências Biológicas e da Saúde, Universidade Federal de Mato Grosso, Pontal do Araguaia, MT 78698-000 Brazil; 6 Departamento de Ecologia, Universidade de Brasília, Brasília, DF 70919-970 Brazil; 7 Integrative Research Institute on Transformations of Human-Environment Systems-IRI THESys, Humboldt-Universitätzu Berlin, Unter den Linden 6, 10099 Berlin, Germany

**Keywords:** Landsat time series, Phenological metrics, Carbon quantification, Savanna, Cerrado, Remote sensing, Random Forest regression

## Abstract

**Background:**

The quantification and spatially explicit mapping of carbon stocks in terrestrial ecosystems is important to better understand the global carbon cycle and to monitor and report change processes, especially in the context of international policy mechanisms such as REDD+ or the implementation of Nationally Determined Contributions (NDCs) and the UN Sustainable Development Goals (SDGs). Especially in heterogeneous ecosystems, such as Savannas, accurate carbon quantifications are still lacking, where highly variable vegetation densities occur and a strong seasonality hinders consistent data acquisition. In order to account for these challenges we analyzed the potential of land surface phenological metrics derived from gap-filled 8-day Landsat time series for carbon mapping. We selected three areas located in different subregions in the central Brazil region, which is a prominent example of a Savanna with significant carbon stocks that has been undergoing extensive land cover conversions. Here phenological metrics from the season 2014/2015 were combined with aboveground carbon field samples of *cerrado* sensu stricto vegetation using Random Forest regression models to map the regional carbon distribution and to analyze the relation between phenological metrics and aboveground carbon.

**Results:**

The gap filling approach enabled to accurately approximate the original Landsat ETM+ and OLI EVI values and the subsequent derivation of annual phenological metrics. Random Forest model performances varied between the three study areas with RMSE values of 1.64 t/ha (mean relative RMSE 30%), 2.35 t/ha (46%) and 2.18 t/ha (45%). Comparable relationships between remote sensing based land surface phenological metrics and aboveground carbon were observed in all study areas. Aboveground carbon distributions could be mapped and revealed comprehensible spatial patterns.

**Conclusion:**

Phenological metrics were derived from 8-day Landsat time series with a spatial resolution that is sufficient to capture gradual changes in carbon stocks of heterogeneous Savanna ecosystems. These metrics revealed the relationship between aboveground carbon and the phenology of the observed vegetation. Our results suggest that metrics relating to the seasonal minimum and maximum values were the most influential variables and bear potential to improve spatially explicit mapping approaches in heterogeneous ecosystems, where both spatial and temporal resolutions are critical.

**Electronic supplementary material:**

The online version of this article (10.1186/s13021-018-0097-1) contains supplementary material, which is available to authorized users.

## Background

Terrestrial ecosystems play a pivotal role in providing regulating ecosystem services related to global and climate change [1]. Photosynthesis and respiration processes of vegetation are the direct link between biosphere and atmosphere, stressing terrestrial ecosystems’ importance in the global carbon cycle [2]. As natural or anthropogenic disturbances such as fires and land cover conversions alter ecosystem functions and eventually can turn carbon sinks into sources, it is crucial to monitor change processes and map related carbon stocks and the changes thereof. A better understanding of the carbon cycle is especially important due to the uncertainties of how vegetation will respond to a changing climate [3, 4]. In addition, accurate quantification of carbon stocks and related changes is essential for measuring and reporting schemes within the context of international climate policies such as the Reducing Emissions from Deforestation and Forest Degradation (REDD+) mechanism of the United Nations Framework Convention on Climate Change (UNFCCD) [3, 5]. National and regional stakeholders also need up-to-date information to support the NDCs and the UN SDGs as a mechanism to finance climate change adaptation related policies [6]. In order to map carbon stocks over large extents remote sensing data have been shown to be mandatory [7, 8]. During the last decades several approaches using active [9–11], passive [12] or both [13] remote sensing data types have proven sufficient accuracies for carbon quantification. This development has been catalyzed by the broad availability of improved remote sensing datasets and the evolution of cutting-edge data mining techniques for remote sensing data analysis [7, 14]. The majority of these studies has focused on dense forest ecosystems, however, a large share of the terrestrial surface is rather characterized by ecosystems with gradual transitions in vegetation density, such as Savannas. Globally, Savannas cover approximately 20% of the land area [15] and even though they usually contain less carbon than dense forest ecosystems they are important carbon sinks [16] and cannot be neglected in global carbon cycle analyses [14, 17]. This is specifically true considering recent trends of land conversions in Savanna regions (e.g. [18]). A prominent example of these ecosystems is the Brazilian Savanna, known as the Cerrado, which covers approximately 2 million km^2^ or ca 23% of Brazil’s surface area [19]. It is characterized by diverse vegetation structure and density, strong seasonality and fire events [20, 21]. The Cerrado is has a high biodiversity with many endemic species [22] and is, due to a weak conservation status and subsequently a loss of habitat, considered as one of the global biodiversity hotspots [23, 24]. Large areas of the natural vegetation have already undergone tremendous land cover changes, leading to a share of approximately 60% of remaining natural vegetation, which is expected to further decline in the future [25]. The combination of these factors directly impacts the link between the land surface and the atmosphere [26], which is e.g. through processes such as photosynthesis and respiration reflected in the vegetation’s phenology [27]. It emphasizes the role of the Cerrado in the carbon cycle, the need for accurate carbon quantifications [28, 29] and for a better understanding of phenology—carbon relations.

Similar to other Savanna regions, the main challenges for remote sensing based carbon quantification in the Cerrado are related to the strong seasonality of rainfall, as cloud cover in the wet season hinders the frequent acquisition of optical imagery [30]. During the last decades, several studies have shown the potential of multi-temporal remote sensing approaches and time series analysis to capture land surface phenology, based on high temporal resolution data from sensors such as e.g. AVHRR [31, 32] or MODIS [26, 33] and also discussed its benefits for biomass estimation [34]. However, approaches based on high temporal resolution data usually lack the spatial resolution that is necessary to monitor heterogeneous and fragmented ecosystems, where reflectance measures are composed of spectral properties from several land cover types [35, 36] and aboveground carbon might change at finer spatial scales than captured in spatial coarse resolution data [12]. Recently, it has been shown that using Landsat data with its spatial resolution of 30 m can help to overcome this shortcoming. Avitabile et al. [12] demonstrated Landsat’s potential for aboveground biomass estimation in Uganda and their results suggest that adding phenological information from multi-temporal imagery could improve model performance by better discriminating vegetation types. In aboveground biomass models of Sudano-Sahelian woodlands, Karlson et al. [37] identified the median of a dry season Landsat NDVI time series as one of the three most important variables. However, Landsat’s relatively low temporal resolution with a revisit time of 16 days challenges deriving annual land surface phenology (LSP) that captures the whole growing season, especially in cloud prone areas [38]. At the same time, the huge amount of freely accessible, archived data holds unexplored potential for ecosystem mapping and monitoring [39].

First promising approaches to analyze dense Landsat time series exist, which are e.g. based on data pooling [35, 40, 41] or gap filling approaches [42, 43]. We here aim to further exploit the approach proposed by Schwieder et al. [43], based on the hypothesis that a link between annual dynamics of vegetation as captured in LSP metrics, the productivity of plants and the aboveground carbon stored in vegetation can be established. Our objectives thus are to (i) investigate the potential to model aboveground carbon in a heterogeneous ecosystem based on Landsat-derived LSP metrics (ii) assess the relation between these phenology metrics and aboveground carbon and (iii) use these metrics to map the carbon distribution across different Cerrado landscapes.

## Methods

### Study areas and field data

The Cerrado stretches from around 2°–25° South. Its elevation ranges from sea level to 1800 m above sea level [19], with most of the Cerrado being part of the Brazilian Central Plateau. Average annual precipitation ranges from 1300 to 1600 mm, with distinct dry (May to September) and wet seasons (October to April). With a mean temperature of 20.1 °C, the Cerrado is classified as Aw climate after Köppen-Geiger, which is typical for Savanna regions [44, 45]. The well drained soils of the Cerrado are mainly dystrophic with rather high aluminum and iron contents [19]. These environmental factors can vary widely over the vast extent of the Cerrado, adding to the heterogeneity of the biome. Fire occurrence and long term climatic fluctuations further increase vegetation variability creating strong gradients in vegetation structure and density over space and time [20]. The resulting mosaic of landscape formations ranges from open grasslands over shrub-dominated areas and scattered tree formations with grassland understory to dense forest patches. This mosaic is therefore classified in distinct physiognomy classes based on their respective vegetation height and tree cover [46]. Different physiognomies are accordingly characterized by different biomass and thus also differ in their shares of stored above- and belowground carbon [16, 47].

**Fig. 1:**
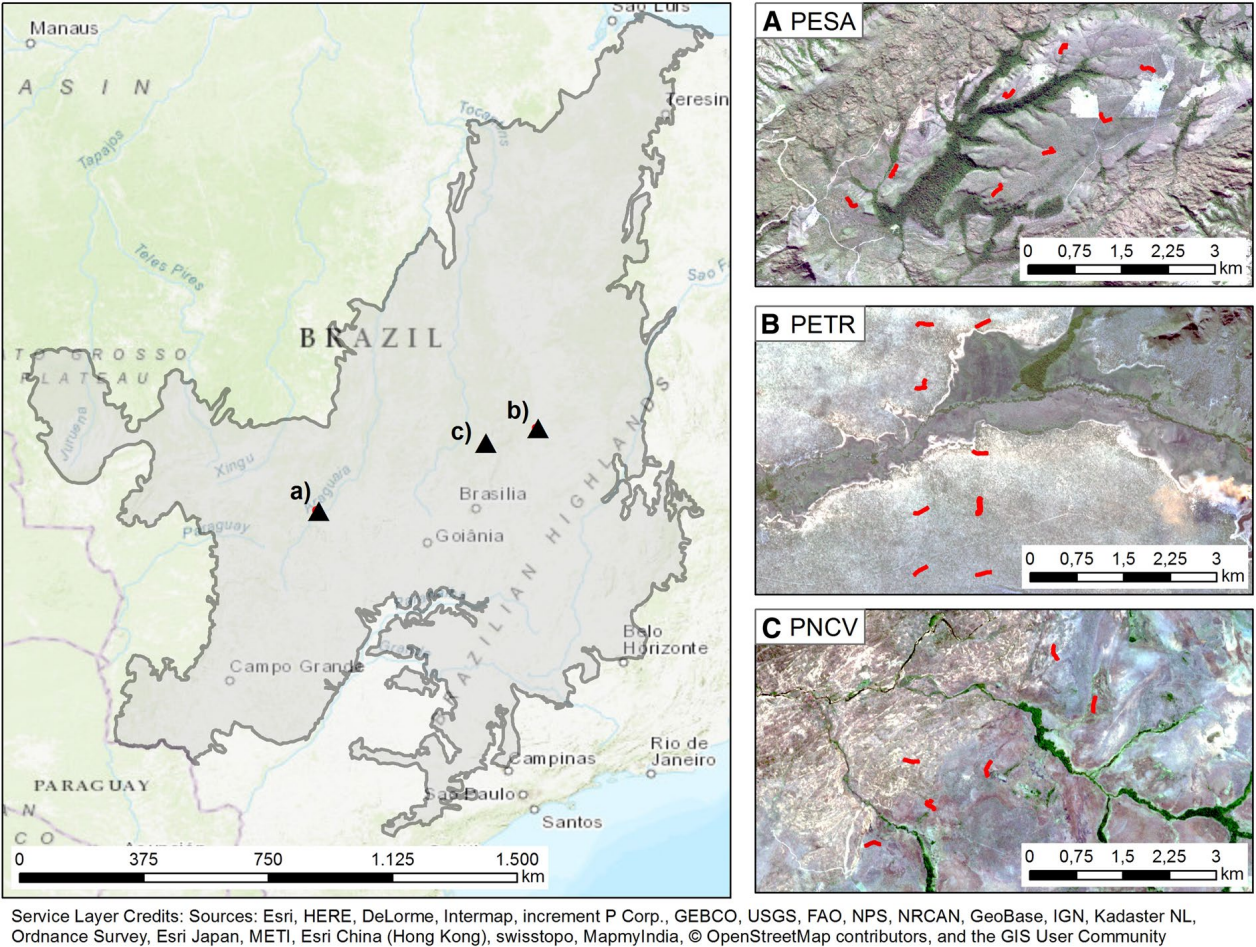
Locations of the three study areas, which are located within **A** Parque Estadual da Serra Azul (PESA). **B** Parque Estadual de Terra Ronca (PETR) and **C** Parque Nacional da Chapada dos Veadeiros (PNCV) in the Brazilian Cerrado. The red polygons show the location of the field transects within the study areas with underlying true color Rapid Eye imagery

This study focuses on three areas in the Brazilian Savanna that are characterized as *cerrado* sensu stricto, which is the most abundant physiognomy in the remaining natural Cerrado vegetation [28]. The *cerrado* sensu stricto may feature 20–50% of tree cover and individual tree heights range from 3 to 6 m [46]. All three sites lie within protected areas, i.e. anthropogenic land cover changes can be neglected for this analysis. Their spatial extents were defined by available field data. The most western of our three study areas is located near the border of the Brazilian federal states of Goiás and Mato Grosso close to the city of Barra do Garças (Fig. 1A; center coordinate: 15.84022 S, 52.23978 W). It lies within the borders of the Parque Estadual da Serra Azul (PESA). According to data from the Shuttle Radar Topography Mission (SRTM) the elevation in our study area ranges between 413 and 771 m asl. The lower elevations are covered by dense semi-deciduous forests, whereas the surrounding higher areas are *cerrado* sensu stricto. The second study area covers parts of the Parque Estadual de Terra Ronca (PETR) (Fig. 1B; center coordinate: 13.62773 S, 46.28531 W). It is located near the city of São Domingos at the border of the states Goiás and Bahia [48]. Here elevations vary between 617 and 1013 m asl. The third study area is located in the North of Goiás state, close to the city of Alto ParaÍso de Goiás within the Parque Nacional da Chapada dos Veadeiros (PNCV; Fig. 1C; center coordinate 14.1046 S, 47.7170 W). Elevations vary from 1068 m to 1267 m asl, with rocky outcrops at higher altitudes. *Cerrado* sensu stricto areas dominate in PNCV with a few gallery forest patches along water bodies [49].

In all three study areas, field plots were established following sampling protocols based on the procedures for the permanent plots of the PPBio program [50] with adaptations to the Cerrado biome. Two parallel 5 km tracks (1 km apart) were defined, with five equally spaced 250 m × 40 m (10,000 m^2^) plots being staked out along each route. The longer dimension of the plots followed the contour of the terrain in order to avoid the possible effects of variations in altitude on its characteristics. Within these plots all trees with a minimum diameter at a height of 30 cm above the ground level of 5 cm were sampled between 2012 and 2014 (taxa, height and diameter). However, we excluded trees with a diameter smaller than 10 cm from the carbon analysis to make sure that we focus on vegetation with relative stable carbon stocks. Field plots that covered vegetation physiognomies other than *cerrado* sensu stricto were excluded from the analysis, resulting in 8 field plots each for PESA and PETR and 6 for PNCV (Fig. 1A–C). Aboveground carbon values for each sampled tree were calculated with a specific allometric equations [51], which is considered representative for the *cerrado* sensu stricto physiognomy as it is based on a broad variety of 174 individuals sampled in Brasília, DF. The resulting carbon values were spatially allocated to the 30 m spatial resolution of the Landsat grid following the approach proposed by Leitão et al. [52]. In the first step the polygons that were sampled in the field are intersected with the Landsat grid. Polygons that fully lie within a Landsat pixel were randomly separated and used to estimate linear regression coefficients based on their individual carbon values measured in the field and a high resolution (5 m × 5 m) RapidEye vegetation index layer (REVI; [53]). The best performing model coefficients were selected based on cross validation. Finally, the REVI layer and the estimated regression coefficients were used to spatially allocate and extrapolate, the carbon values measured in the field, to the unsampled areas of the intersecting Landsat pixel [52].

### Phenological metrics

**Fig. 2:**
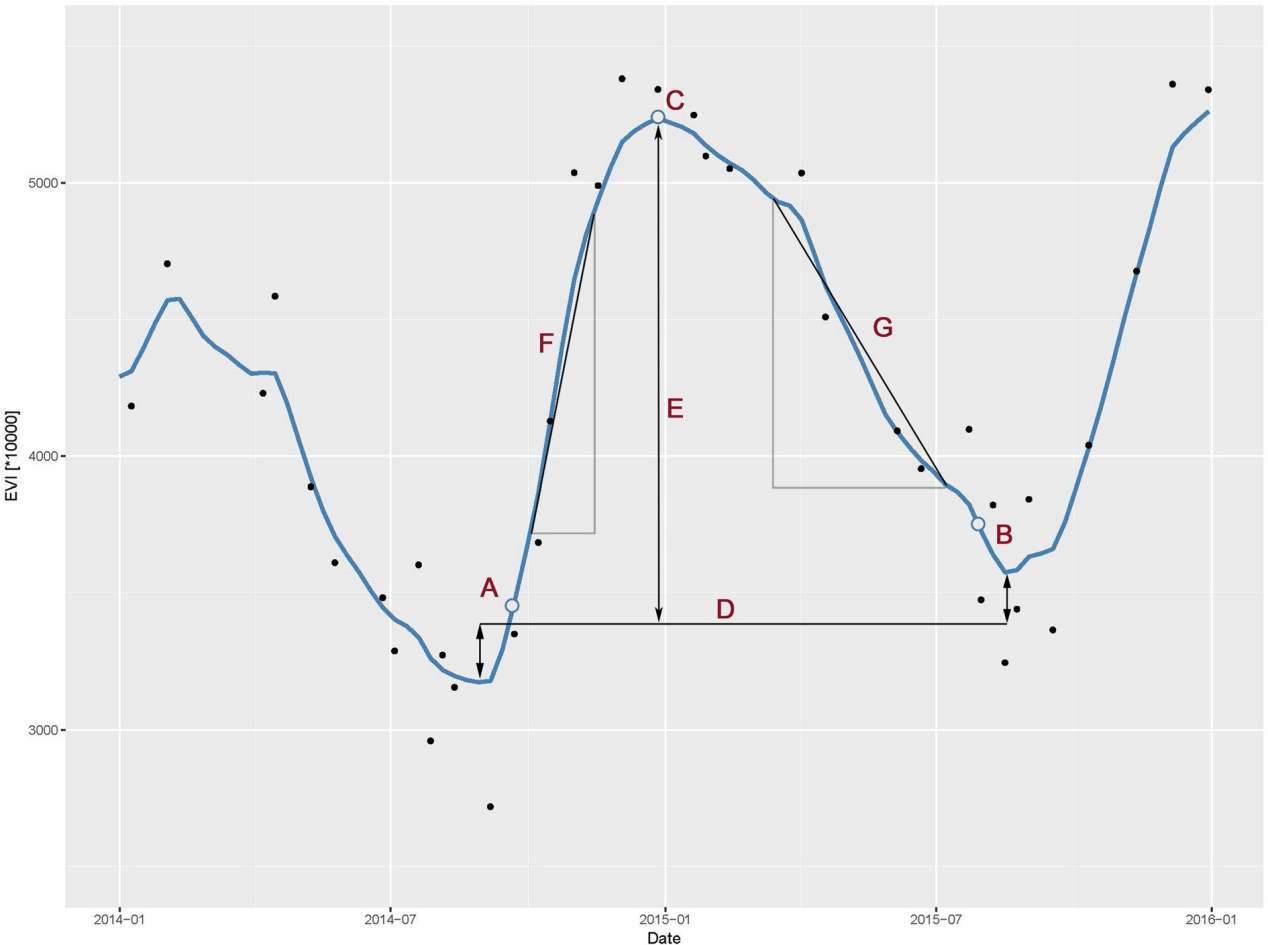
Phenological pixel profile in PESA (-15.851089; -52.261512) after outlier detection and RBF fitting, along with in TIMESAT derived phenological metrics (*A*: start of season; *B*: end of season; *C*: maximum fitted value; *D*: base value; *E*: amplitude; *F*: rate of increase; *G*: rate of decrease). The black points represent the original Landsat EVI values and the blue line the fitted RBF ensemble values within 8-day temporal bins

Land surface phenology and related phenological metrics were derived from a combined Landsat ETM+ and OLI 8-day time series. Therefore, all available L1T corrected Landsat ETM+ and OLI surface reflectance data (path/row: PESA 224/071; PNCV 221/070; PETR 220/069) that were acquired between the beginning of 2014 and the end of 2015 (cloud cover < 90%) were downloaded along with their respective cfmask product [54]. The enhanced vegetation index (EVI) was calculated based on Eq. (1), as it is known to decouple the canopy background signal and to reduce atmospheric influences, while still being sensitive to high biomass/carbon values [55]:

1 $$\varvec{EVI} = 2.5\varvec{* }\frac{{\varvec{\rho NIR } - \varvec{ \rho RED}}}{{\varvec{\rho NIR } + \varvec{ }6\varvec{ * \rho RED} - 7.5\varvec{ * \rho BLUE } + \varvec{ }1}}$$

where $\varvec{\rho}$ relates to the surface reflectance values in the respective Landsat bands covering the near infrared (NIR), red (RED) and blue (BLUE) wavelengths of the electromagnetic spectrum. Following Schwieder et al. [43], a weighted ensemble of three radial basis convolution filters (RBF) with varying kernel widths (σ) was used to fill temporal data gaps in a vegetation index time series at pixel level. The RBF ensemble was applied, using temporal bins of 8 days, to the period from the first of January 2014 to the 31 of December 2015, resulting in a total of 92 potential original Landsat observations within 2 years, to which available original data were assigned based on their respective acquisition dates. Outliers were excluded from the time series if they were more distant than one standard deviation to a convolution filter function with a kernel width of σ = 20 [43]. Three convolution filters with kernel widths of σ = 8, σ = 16 and σ = 32 were subsequently used to fill the data gaps. The final time series profile is the combination of the three, whereas each filter is weighted based on the original data availability (Fig. 2). To assess the deviation of the fitted RBF values from the original Landsat ETM+ and OLI EVI values, the RMSE was calculated for each sample pixel. Finally, the gap-filled time series were further processed in TIMESAT [56] to derive LSP metrics (Fig. 2). As it was not expected to detect more than one phenological season in the natural vegetation of the Cerrado, the seasonality parameter was set to 1 and the start/end of season were defined as the day of year when 20% of the seasons amplitude was reached from the left (start of season) or right (end of season) minimum of the seasonal profile. Following the detection of start of season (SoS) and end of season (EoS), the length of Season (LoS) was derived as the difference between the two variables. A further variable that relates to the timing of the seasons is the mid of season (MoS). It was derived as the average day of year when the respective 80% level right and left side of the peak has been reached. The Base value (BV) was calculated as the mean EVI value of the two seasonal minima and the maximum fitted value (MfV) is the largest EVI value of the fitted time series. Amplitude (Amp) relates to the range of EVI values between BV and MfV. The rate of increase (RoI) and Rate of decrease (RoD) are calculated as the ratio between the difference in EVI at 20 and 80% levels of the left (RoI) and right (RoD) side of the peak and the corresponding difference in time in absolute values. For further details on the derived metrics see Jönsson and Eklundh [56].

### Carbon model

To map carbon distributions as well as to analyze the relation between phenological parameters and aboveground carbon values, we used a random forest regression (RFR). RFR has been shown to adequately estimate carbon from remote sensing data that is usually not linearly related to metrics from satellite imagery [57]. RFR is an ensemble approach, which is based on the Classification and Regression Tree algorithm [58]. Best splits of the training data are derived at each node using a subset of the input features. As single trees are assumed to be prone to errors, RFR builds many regression trees (i.e. a forest) from random subsets of the input data and validates the results on the withheld data. The final result of the regression is the averaged outcome of all regression trees [59].

The carbon model for each study area was iterated 1000 times with randomly drawn subsets of 70% training and 30% validation data to derive performance measures and to obtain statistically robust results. Model performance was assessed using the root mean square error (RMSE), the relative root mean square error (relRMSE), defined as the ratio between RMSE and the mean trainings pixel carbon values, as well as the coefficient of determination (R^2^). To derive the optimum number of sample pixels, considering the area of the pixel that was actually measured in the field a sensitivity analysis was performed for each study area [52]. Therefore, we executed our carbon models with subsets of the original sample data set based on the percentage of pixel area sampled in the field in 10% steps. The individual sample sets for each study area were subsequently filtered for further analysis, using the derived optimal thresholds. The final maps are based on the mean carbon predictions after 1000 iterations. To further assess the relation between the phenological metrics and aboveground carbon, we evaluated the influence of the individual variables based on the RFR variable importance. It is derived by calculating the difference between the cross-validated model performance (out-of-bag mean square error; MSE) using all variables as model input and the performance of a model with permutated values within the respective variable, which enables a ranking of the most important variables by increase in MSE. The measures are scaled based on their respective standard errors [60]. Partial dependency plots (PDP) of the phenological metrics were created using the R-package pdp [61]. These plots allow analyzing the influence of each input variable on the response, by individually evaluating the Random Forest model based on the variations within one selected variable, while all other variables are fixed to their respective mean. However, as PDP’s are useful to analyze the relation between carbon distribution and LSP metrics, but do not reveal the relations among the input variables, a principle component analysis (PCA) was performed based on the correlation matrix of the phenological metrics. Then the carbon values were plotted within the new feature space. Both analyses were derived for three RFR models based on all available samples of each study area, considering the respective sensitivity analysis threshold. The carbon models were built in the R environment [62] using the tuneRF function of the random Forest R-package [60] for automated model parameter optimization.

## Results

During the season of interest (2014/15) a total of 46 Landsat ETM+ and OLI observations were acquired at 8-day intervals. Cloud coverage and sensor errors led to a reduced effective observation density in our study areas, which greatly differed between the dry and the wet season. On average, 23 (PESA), 21 (PNCV) and 26 (PETR) observations of our sample pixels were available for the whole season. During the dry season, an average of 13 (PESA), 16 (PNCV) and 17 (PETR) from 20 potential observations were available, in contrast to 10 (PESA), 5 (PNCV) and 9 (PETR) from 26 during the wet season (Table 1). The deviations between the fitted and the original EVI values resulted in an average RMSE of 0.018 in the PESA sample pixels, where fitted EVI values ranged around a mean of 0.344. Based on the PNCV samples the average RMSE was 0.010 and the fitted RBF EVI values had a mean of 0.238. In PETR the RMSE was 0.013 with a fitted EVI mean of 0.271. The mean allocated aboveground carbon values were 5.47 t/ha in PESA, 3.66 t/ha in PNCV and 4.73 t/ha in PETR (Table 1). From these dense 8-days Landsat time series pixel-wise seasonal phenological metrics were derived for each study area, whenever TIMESAT recognized a full season. These metrics, which describe the course of the phenological profiles, enabled a standardized interpretability of the results and reduced the amount of model input variables from 46 EVI values to 9 phenological metrics per pixel.

**Table 1:** Average data availability from Landsat ETM+ and OLI observations within the sample pixels for the dry (May–September 2014) and wet (October 2014–April 2015) season, relative to the amount of potentially available original observations

	No. of samples	Data availability dry season [%]	Data availability wet season [%]	Mean RMSE (min; max)	Mean RBF EVI (min; max)	Mean allocated carbon [t/ha] (min; max)
PESA	198	63	39	0.018 (0.012; 0.024)	0.344 (0.204; 0.447)	5.47 (0; 15.21)
PETR	207	85	36	0.013 (0.007; 0.018)	0.271 (0.176; 0.350)	4.73 (0; 20.56)
PNCV	165	81	18	0.010 (0.005; 0.016)	0.238 (0.183; 0.299)	3.66 (0; 14.63)

The mean RMSE values are based on the deviations between the fitted and the available original EVI values for each study area

The spatial allocation approach, which was used to match field polygons and the phenological metrics pixel grid, led to a total of 198 sample pixels in PESA and 207 in PETR to be used as input for the carbon models. As some of the data in the 165 PNCV pixel were too noisy to derive phenological parameters, 15 pixels were excluded from the carbon models. The regression coefficients used for spatial allocation were 2.810 (PESA), 3.973 (PNCV) and 2.695 (PETR).

**Fig. 3:**
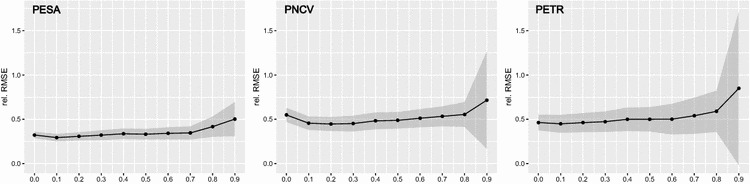
Results of the regression model sensitivity analysis for each study area. Relative RMSE after 1000 model runs are shown for each threshold, while the grey ribbons relate to ± one standard deviation

The sensitivity analysis revealed that carbon model performance generally decreased and was less stable during 1000 iterations, when fewer samples were included in the model (Fig. 3). In terms of relative RMSE the models performed best with thresholds of around 0.1. Thus all pixels in which less than 10% was sampled in the field were excluded from further analysis.

Carbon model performances differed between the three study areas with averaged R^2^ values of 0.69 for PESA, 0.43 for PETR and 0.36 for PNCV and a similar trend in RMSE values of 1.65 t/ha (relative RMSE 0.30) in PESA, 2.18 t/ha (relative RMSE 0.45) in PETR and 2.35 t/ha (relative RMSE 0.46) in PNCV (Table 2).

**Table 2:** Averaged model performance measures (R^2^ and RMSE) and related standard deviations after 1000 iterations, along with the average descriptive statistics of the carbon measures (t/ha)

	Threshold	Number of samples	Mean R^2^	R^2^std	Mean RMSE	RMSE std	Mean relRMSE	Carbon min	Carbon mean	Carbon max
PESA	0.1	145	0.70	0.06	1.64	0.20	0.29	0.44	5.59	14.92
PETR	0.1	163	0.44	0.14	2.18	0.44	0.45	1.07	4.88	19.71
PNCV	0.1	90	0.36	0.11	2.34	0.34	0.46	0.42	5.16	14.22

**Fig. 4:**
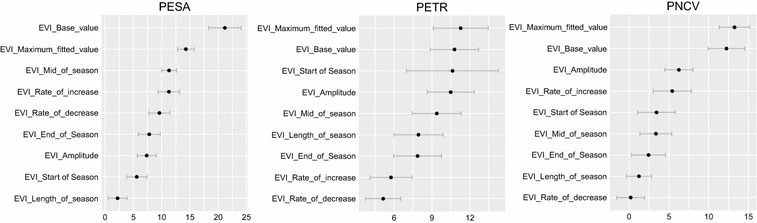
Mean variable importance measures for the three study areas after 1000 model iterations. The values are scaled using their respective standard errors. Horizontal bars indicate standard deviations

Despite the differences in model performance, the variable importance ranking was largely stable across models, with BV and MfV being ranked as first or second most influential variables across all study areas (Fig. 4). Ranks of further variables varied between the study areas, with higher standard deviations in PNCV and PETR. Especially in PETR the individual variable importance was small with comparably high standard deviations (Fig. 4).

**Fig. 5:**
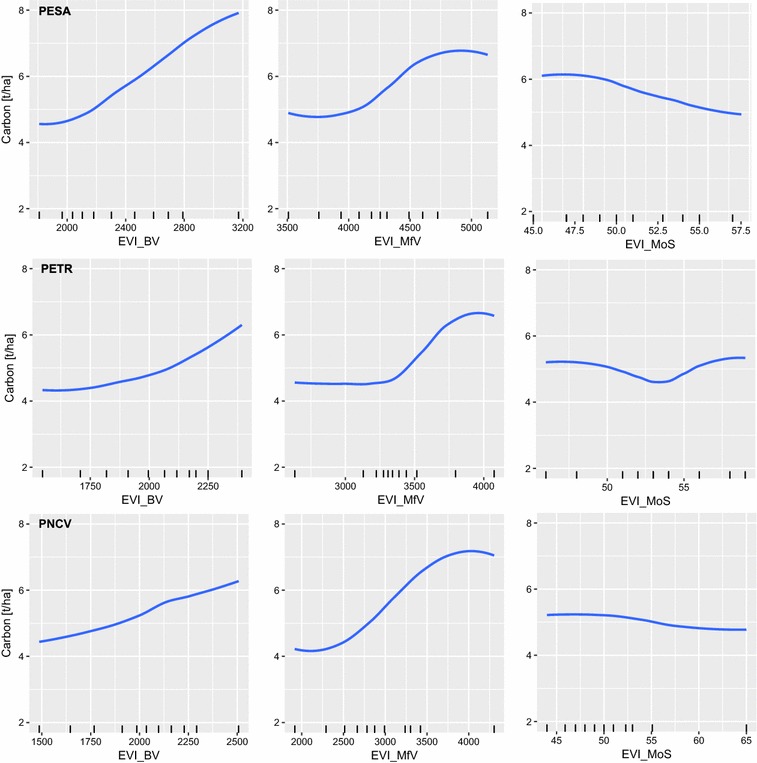
Selected smoothed partial dependency plots (PDP) for each study area. Plots are derived by validating a RFR model using all available samples of each study area. The additional ticks on the x-axis mark the min/max and decile values of the input variable. BV and MfV are shown in EVI * 10,000, MoS are shown as 8-day temporal bins starting from 01/01/2014. PDP’s of all variables are shown in the Additional files 1, 2, 3

The partial dependency plots of important phenological metrics (Fig. 5) highlight the relation between the phenological metrics and carbon distributions. BV and MfV are positively correlated to carbon values throughout all study areas. While BV steadily increases with increasing carbon values even in data sparse regions, saturations occur in MfV when carbon values approximate around 5.7–6.7 t/ha. Even though carbon value distributions vary among the study areas, the relation between BV and carbon is similar. The relation between MoS and carbon is comparable in PESA and PNCV with lower carbon values being associated to a later peak of season. Even though the trend is not as clear in PETR, it remains comparable to the other regions until mid of season around DOY 54.

**Fig. 6:**
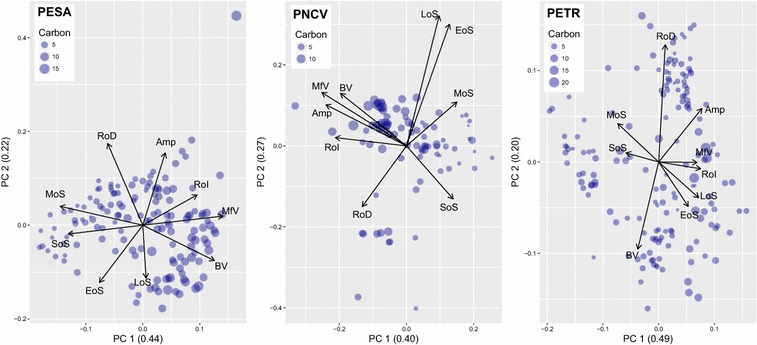
Plot of the first two axes of the PCA of the phenological metrics for each study area (numbers in brackets report the explained variance within the respective principal component). The angle between the arrows depict approximately the correlation between the phenological metrics. The points mark the carbon values within the new variable ordination space, while their size refers to the original carbon values in t/ha

Additional to the analysis of the relations between carbon and individual phenological metrics, the principal component analysis (PCA) reveals the distribution of carbon values within an uncorrelated variable space (Fig. 6). In PESA the first two axes explain 66% of the variance within the phenological metrics. The first axis is defined by EVI-related values such as BV and MfV, as opposed to metrics related to the timing of the season such as MoS and SoS. The latter are related to smaller carbon values, whereas BV and MfV are associated with larger values. The second axis is defined by the negative correlation between temporal metrics (EoS and LoS) and EVI related values (RoD and Amp). In PNCV the first two axes of the PCA explain 67% of the metrics’ variation. Here the negative correlation between EVI metrics (Amp, BV, MfV) and seasonal timing (SoS) is revealed, where a cluster of rather large carbon values is oriented towards MfV, BV and Amp. The second axis is defined by the negative correlation between LoS, EoS and RoD, where average carbon values cluster. In the case of PETR, the first two axes of the PCA explain 69% of the variance. The first axis is defined by the temporal metrics MoS and SoS and their negative correlation with RoI and MfV, with the latter being associated to rather larger carbon values. The second axis is defined by RoD on one side and BV on the other side, with rather equally distributed carbon values at both ends.

**Fig. 7:**
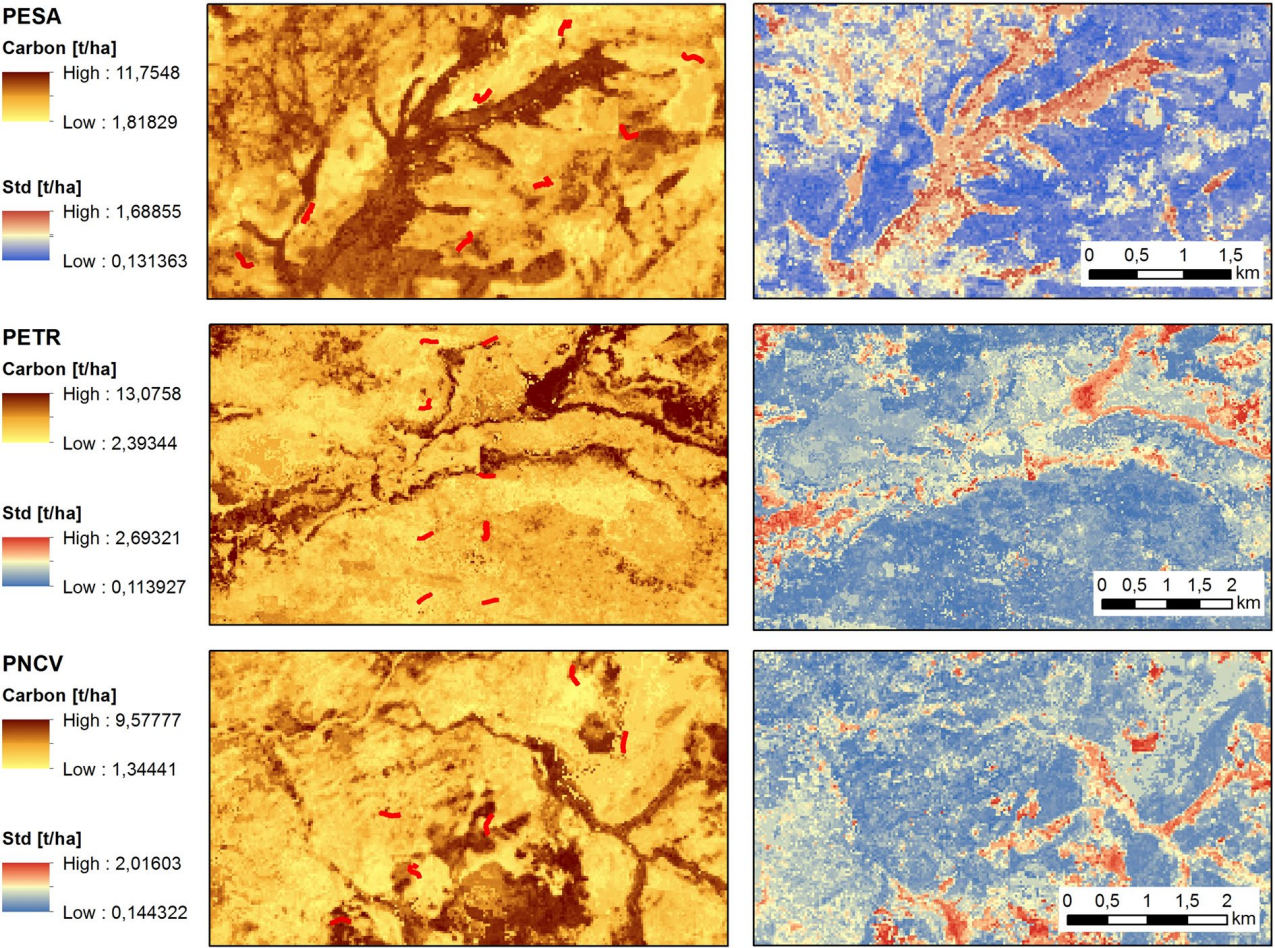
Left: Carbon maps for the study areas PESA, PNCV and PETR based on the mean predictions of 1000 individual Random Forest regression models for each study area along with the sampling transects in red. Right: Corresponding standard deviation maps

The carbon distribution was mapped based on the mean of 1000 model predictions throughout the selected study areas (Fig. 7). The final RF models explained 68% (PESA), 37% (PETR) and 49% (PNCV) of the withheld variance on average. The predicted carbon values range between 1.8 and 11.8 t/ha in PESA, between 2.4 and 13.1 t/ha in PETR and between 1.3 and 9.6 t/ha in PNCV. Standard deviations of up to 1.7 (PESA), 2.7 (PETR) and 2.2 (PNCV) mainly relate to predictions for areas with high carbon values.

## Discussion

Spatially explicit quantification of aboveground carbon distributions is essential to monitor and understand ongoing changes in carbon stocks. In heterogeneous landscapes, such as typical for the Cerrado, the spatial and temporal resolution at which we monitor the ecosystem play a pivotal role for our ability to map carbon. The presented results highlight the potential of annual LSP metrics, derived from dense 8-day Landsat time series with a spatial resolution of 30 m × 30 m, for the spatially explicit quantification of aboveground carbon. Using all available satellite observation from Landsat enabled us to assign field based carbon values to LSP metrics from Landsat time series that describe the seasonal changes of the monitored vegetation as captured in the EVI.

Although the regression model performances vary between the three observed study areas they are still within a range that is comparable to results from studies dealing with the mapping of carbon in similar ecosystems. For example, González-Roglich and Swenson [17] used, among other variables, products derived from Landsat data to map carbon distributions in Argentinian Savanna regions. Their spatially explicit predictions, based on field samples ranging around a mean of 27 t/ha, had a mean prediction error of 9.6 t/ha (relRMSE 35%) at 60 m spatial resolution. Karlson et al. [37] reported model performances with an R^2^ of 0.57 and RMSE of 17.6 t/ha (relRMSE 66%) when mapping aboveground biomass in Sudano-Sahelian woodlands using multi-temporal (7 observations) Landsat OLI products. Remote sensing based carbon estimations are prone to several sources of errors that propagate through the individual steps of the study, thus influencing the final model uncertainty. Errors may be introduced, on the part of the field sampling, by the defined plot size and sampling design, measurement errors during field work, allometric equations (to derive carbon values) and the spatial allocation of field samples to the pixel grid. However, according to Chave et al. [63] the main source of error can be attributed to the chosen allometric equation. Even though the allometric model that we employed is representative for the most abundant plant families within the selected study areas (*Vochysiaceae* and *Fabaceaea*), we found differences between the three study areas in species composition and abundance. Especially, species that were prominently abundant in PNCV and PETR (e.g. v*ellozia squamanta, virtella ciliata*) were not considered for deriving the allometric model presented in Rezende et al. [51] and might explain variations in the overall model performances. Even though the magnitude of the influence could not be quantified it stresses the importance for refined allometric equations in further research. Another source of uncertainty is the chosen sampling design and the associated sampling plot size. We accounted for unsampled pixel regions using a sophisticated spatial allocation approach, which employed high-resolution reference data as a weighting layer [52] and evaluated the trade-off between total sampling size and extrapolated pixel values. It could be observed that a decrease in the number of samples led to weak and less robust model performances, i.e. an increase in relRMSE and the related standard deviations. Nevertheless, we did not quantify the influence of the general plot sampling size on the final model outcome. A meta-study by Zolkos, Goetz [64] compared model performances of 70 + biomass estimation studies (based on Lidar, Radar, optical and combined data sets) and revealed the influence of the sampling plot size on model performance. Their comparison revealed relative residual standard errors of biomass models with plot sizes below 0.2 ha (Landsat pixel ≙ 0.09 ha) that range between 10 and 50% [64]. Chave et al. [63] estimated uncertainties due to plot sizes of 0.1 ha to be > 10% and suggest at least 50 × 50 m plots to represent study site variability. However, as the employed sampling strategy was not solely intended for carbon estimations but for a range of ecological research, it was designed to meet several criteria. The sampling strategy might thus not have been sufficient to equally capture the respective study site heterogeneities regarding the regional carbon distribution (see Additional file 4 for histograms). Our results show that the spatially allocated carbon values differ between the three study areas with highest values in PESA, followed by PETR and PNCV. This trend is also reflected in the distribution of EVI values and the respective phenological metrics and follows the gradient from overall lower (PESA) to higher (PNCV) elevations above sea level between the observed areas.

Along with the abovementioned findings our results stress the complexity and challenges of carbon mapping approaches, especially in heterogeneous Savanna systems, in which an additional critical issue is the influence of understory vegetation (grasses and shrubs). Our samples only considered woody vegetation with a diameter of at least 10 cm. Thus, the carbon values lack shares of smaller trees and the non-woody vegetation layer, which would require a more frequent field sampling design to capture their dynamics. Even though the below canopy vegetation is not considered in the field sample, it is still influencing the spectral signal throughout a season. We accounted for these variations by interpreting EVI based LSP metrics that describe a full annual season, as EVI values are known to be sensitive to structural variations in vegetation canopies and the canopy background is decoupled from the signal [55]. Our results indicate that the RBF filter ensemble was able to capture the seasonal profile of the original observations, with minor deviations from the original EVI values. Using all available cloud-free data from Landsat provides a maximum number of observations as the basis for fitting the RBF and accordingly data gap fill values represent the best possible temporal interpolation. But even though the RBF ensemble approach accounts for data availability through adjusted weights, the variations in available observations between the dry and wet season still have an influence on the phenological profiles and ultimately the derived metrics. This is a limitation that in the future is likely to be overcome by the integration of additional data from other sensors, such as Sentinel-2 [65].

Despite the discussed restrictions in our models, we showed, to our best knowledge for the first time, the benefits of using LSP metrics with a 30 m × 30 m spatial resolution for carbon modelling, contributing to the body of research that employ LSP metrics for carbon estimations (e.g. [66–68]). Our results highlight that in contrast to raw time series of vegetation indices, LSP metrics simplify further analysis of the relation between aboveground carbon and LSP.

Especially metrics that are directly related to the amount of vegetation, such as base value and maximum fitted value, have the potential to explain carbon variations, as they were ranked as first or second most important in our regression models. This pattern is also revealed by the PCAs that on the one hand show correlations among the LSP metrics but also reveal relationships between them and carbon values. In all three study areas, but especially prominent in PESA, are clusters of lower carbon values associated to high values of variables related to seasonality, such as SoS or MoS. This suggests that vegetation with higher carbon densities might be related to an earlier start of season, which is here defined as the point in time when 20% of the ascending part of the phenological profile is reached, as well as an earlier peak of season. A possible explanation might be the leaf producing strategy of some of the (semi-) deciduous species, for which a high activity of leaf production could be observed at the end of the dry season [69, 70], causing an earlier green-up in the phenological profile. Similar phenological patterns have been observed in the western part of the Sudanian Savanna, where a later start of season was observed in LSP for areas with higher shares of herbaceous than woody vegetation [71].

The final carbon maps show comprehensive spatial patterns with e.g. high carbon values along the riparian vegetation (gallery forests) and the dense forest patches in the lower elevations in PESA. Vegetation patterns in very high resolution imagery from RapidEye suggest that the region’s heterogeneity is very well reflected in the spatial variations of the mapped carbon patterns. The spatially explicit quantification captures the landscape composition and highlights the benefits of Landsat’s spatial resolution for estimating carbon across different study areas in the Cerrado. This is also stressed when comparing the final carbon maps to available wall-to-wall carbon products such as the high-resolution carbon map for Brazil [72] or the carbon base map of the year 2000 presented by Zarin et al. [73]. However, these products are based on a range of input data with varying spatial resolutions, aiming to estimate carbon distributions of woody vegetation with a global model. A direct comparison of these carbon values to our regional model outputs is therefore not straight-forward, but can be used to assess the agreement between spatial patterns.

The range of mapped carbon values is in line with values found in the literature. Carbon values for cerrado sensu stricto are for example summarized in Ribeiro et al. [29], as well as in Vourlitis and da Rocha [74] and range between 3.3 and 32.5 t/ha (mean 8.5 t/ha) or 5.0–15.9 t/ha (mean 9.7 t/ha), respectively, depending on the regional focus and the methods used. However, due to a lack of additional reference data, the maps could not be independently validated and especially estimates for physiognomies that were not included in the training samples (such as grasslands and dense forests areas) need to be regarded with caution. Indeed, the carbon-dense areas, e.g. gallery forests and forest patches (e.g. seasonal forest and Cerradão), are associated with the highest standard deviations, as they are model extrapolations and our models will most likely underestimate carbon stocks in these areas. Our results highlight that phenological metrics derived from freely available remote sensing data are a valuable contribution to carbon mapping approaches, providing spatially explicit knowledge for environmental managers and policy makers in support of sustainable development policies related to REDD+ or the UN SDG’s.

## Conclusions

Based on gap-filled 8-day Landsat EVI time series, we derived annual land surface phenology (LSP) metrics for *cerrado* sensu stricto vegetation. The derived metrics enabled us to reduce the amount of input data for a following Random Forest regression analysis, while preserving the information needed to approximate LSP. They further facilitated determining a relationship to the aboveground carbon distribution, with an adequate spatial resolution for mapping gradual vegetation transitions in heterogeneous Savanna ecosystems, such as the Cerrado. Our results are comparable to those of similar studies and we successfully identified the relation between the seasonal behavior of *cerrado* sensu stricto vegetation and its carbon distribution. Metrics that are instantly linked to amounts of vegetation such as Base value and Maximum fitted value have been shown to be important for such a mapping approach. Metrics relating to the timing of phenological events, such as start or mid of season showed a weaker relation and were not consistently relevant for carbon mapping. We were able to map carbon distributions within the selected study areas, whereat higher uncertainties were identified in physiognomies and related carbon values that were not well represented by the field sampling. We verified that Landsat based annual LSP metrics are beneficial variables to analyze carbon—phenology relations and for the spatially explicit quantification of aboveground carbon in heterogeneous ecosystems such as the Cerrado. In order to improve large-scale carbon mapping efforts, our findings stress the need for representative sampling strategies, along with subsequent improvement of allometric equations, which together reflect the variability within the observed Savanna vegetation gradient. Further research should investigate the potential of mapping approaches that synergistically combine phenological metrics with variables related to the vertical structure of vegetation (such as lidar or radar) and analyze the influence of additional data (e.g. Sentinel-2) on the accuracy of the derived phenological metrics.

## Additional files

**Additional file 1.** All partial dependency plots for PESA (Serra Azul State Park, Brazil) for RFR models based on all available samples using the threshold 0.1. Metrics that relate to index values are shown in EVI * 10,000. Metrics related to time are shown as 8-day temporal bins starting from 01/01/2014.

**Additional file 2.** All partial dependency plots for PETR (Terra Ronca State Park, Brazil) for RFR models based on all available samples using the threshold 0.1. Metrics that relate to index values are shown in EVI * 10,000. Metrics related to time are shown as 8-day temporal bins starting from 01/01/2014.

**Additional file 3.** All partial dependency plots for PNCV (Chapada dos Veadeiros National Park, Brazil) for RFR models based on all available samples using the threshold 0.1. Metrics that relate to index values are shown in EVI * 10,000. Metrics related to time are shown as 8-day temporal bins starting from 01/01/2014.

**Additional file 4.** Histograms of the carbon distribution in the three study sites after the spatial allocation of field samples to the pixel grid.

## Abbreviations

Amp: amplitude
Asl: above sea level
AVHRR: advanced very high resolution radiometer
BV: base value
EoS: end of season
EVI: enhanced vegetation index
LoS: length of season
LSP: land surface phenology
MfV: maximum fitted value
MODIS: moderate resolution imaging spectroradiometer
MoS: mid of season
NDC: Nationally Determined Contributions
PCA: principal component analysis
PDP: partial dependency plot
PESA: Parque Estadual da Serra Azul
PETR: Parque Estadual de Terra Ronca
PNCV: Parque Nacional da Chapada dos Veadeiros
RBF: radial basis convolution filter
REDD+: Reducing Emissions from Deforestation and Forest Degradation
relRMSE: relative root mean square error
REVI: RapidEye vegetation index
RFR: random forest regression
RMSE: root mean square error
RoD: rate of decrease
RoI: rate of increase
SDG: Sustainable Development Goals
SoS: start of season
Std: standard deviation
UNFCCD: United Nations Framework Convention on Climate Change
