# Supplementary figures and images for: Landsat phenological metrics and their relation to aboveground carbon in the Brazilian Savanna
Source: Carbon Balance Manag. 2018 May 15;13:7. doi: 10.1186/s13021-018-0097-1 (PMC5953907; doi:10.1186/s13021-018-0097-1)

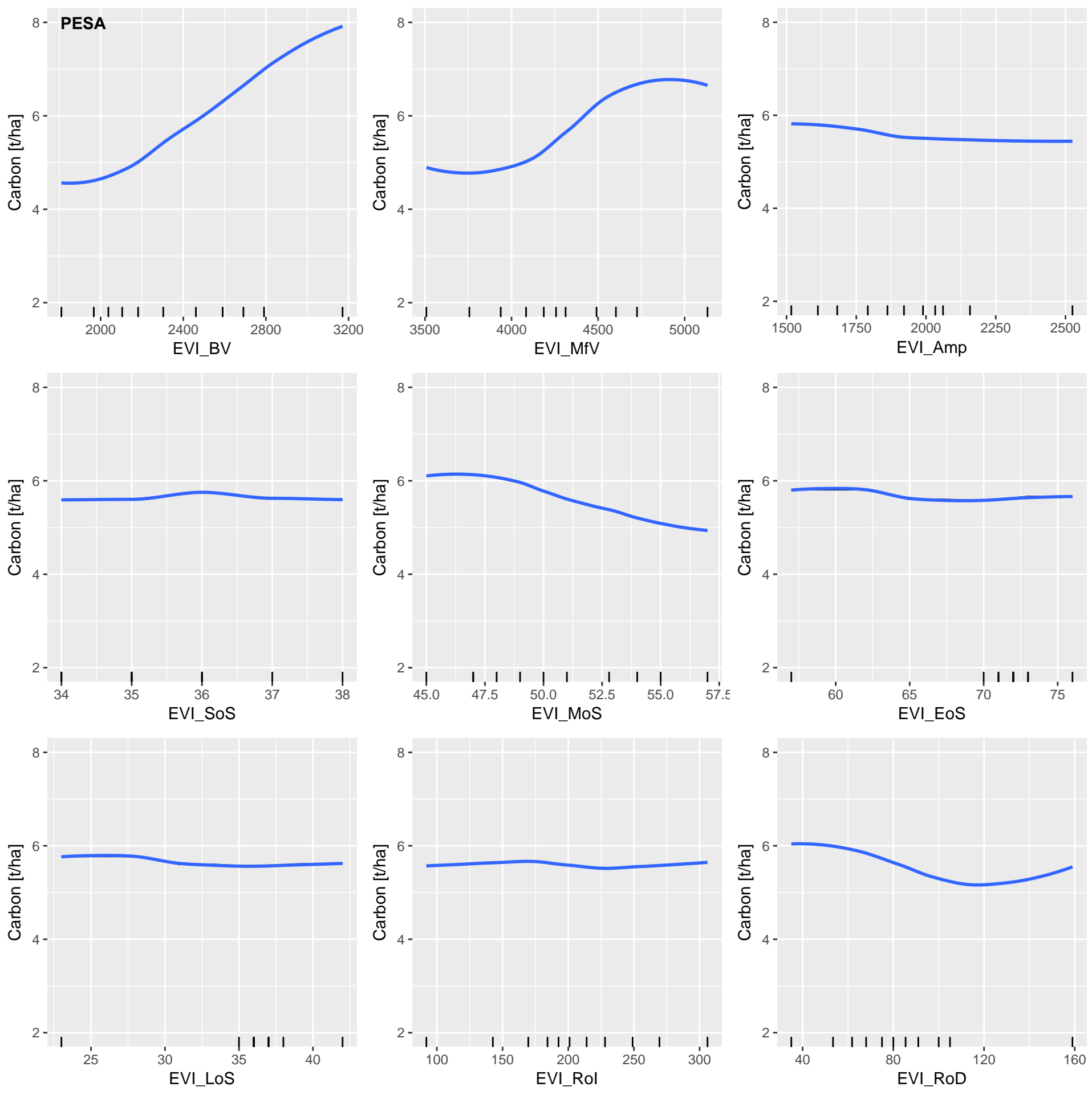

Supplement: Supplementary file 1 — Additional file 1. All partial dependency plots for PESA (Serra Azul State Park, Brazil) for RFR models based on all available samples using the threshold 0.1. Metrics that relate to index values are shown in EVI * 10,000. Metrics related to time are shown as 8-day temporal bins starting from 01/01/2014. [file 13021_2018_97_MOESM1_ESM.pdf]

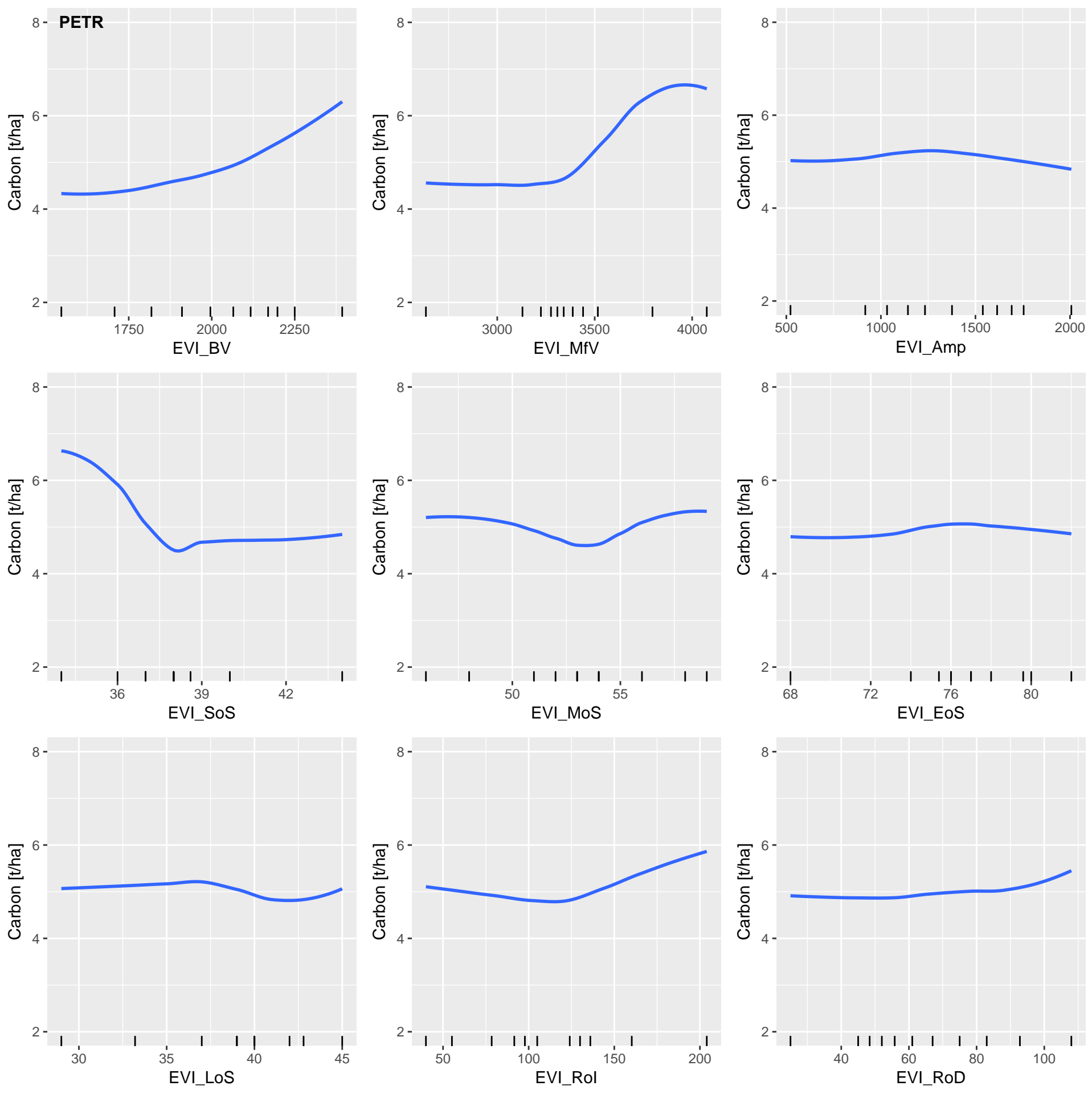

Supplement: Supplementary file 2 — Additional file 2. All partial dependency plots for PETR (Terra Ronca State Park, Brazil) for RFR models based on all available samples using the threshold 0.1. Metrics that relate to index values are shown in EVI * 10,000. Metrics related to time are shown as 8-day temporal bins starting from 01/01/2014. [file 13021_2018_97_MOESM2_ESM.pdf]

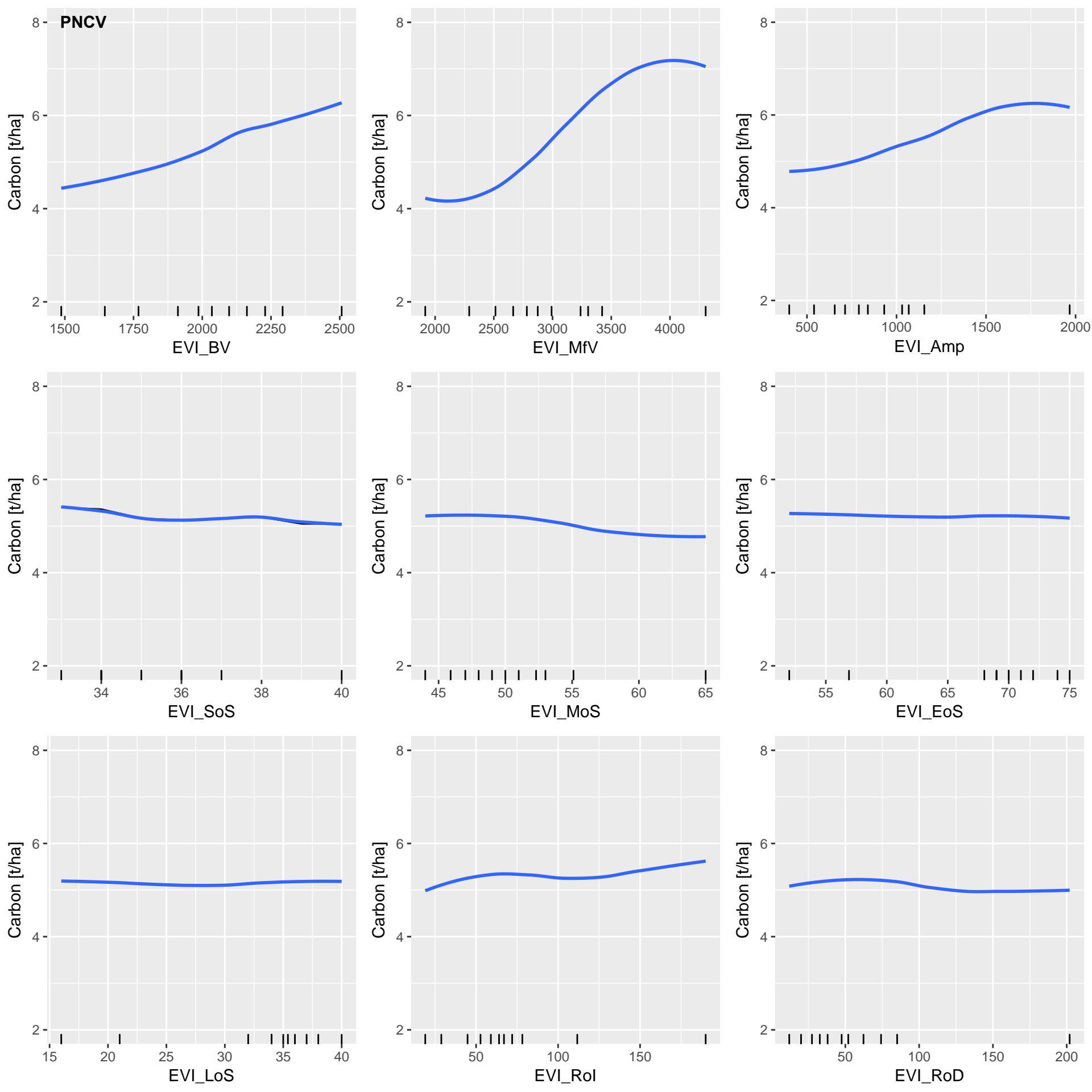

Supplement: Supplementary file 3 — Additional file 3. All partial dependency plots for PNCV (Chapada dos Veadeiros National Park, Brazil) for RFR models based on all available samples using the threshold 0.1. Metrics that relate to index values are shown in EVI * 10,000. Metrics related to time are shown as 8-day temporal bins starting from 01/01/2014. [file 13021_2018_97_MOESM3_ESM.pdf]

PESA

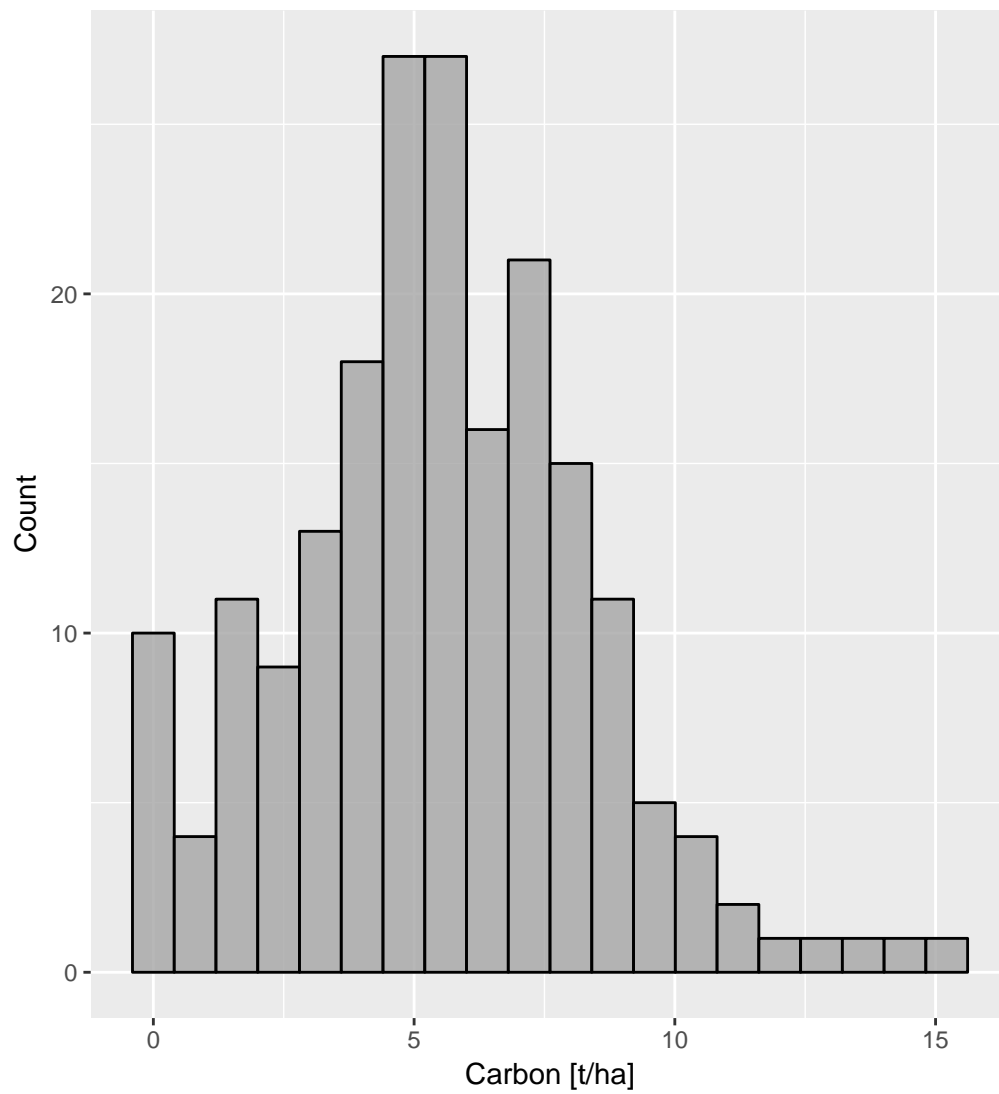

PETR

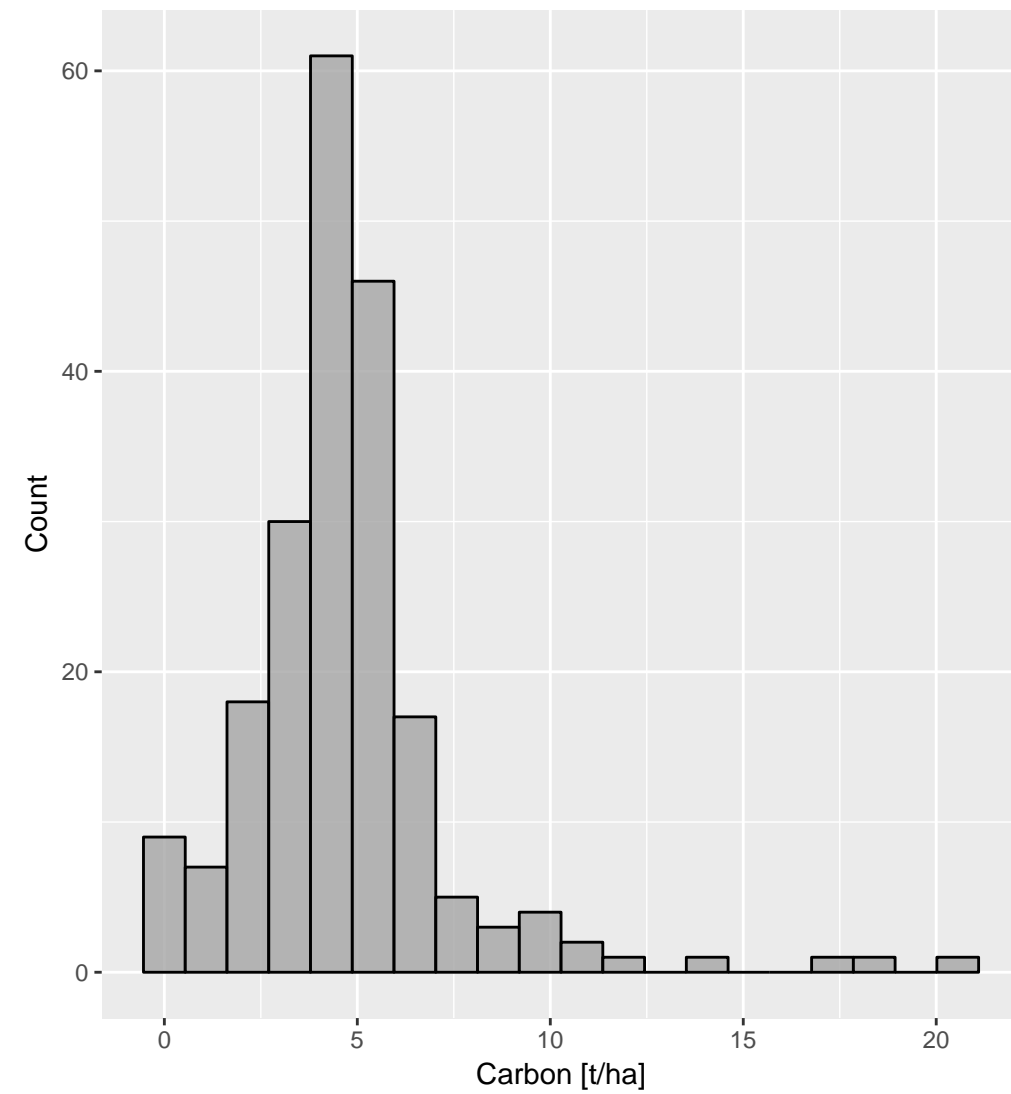

PNCV

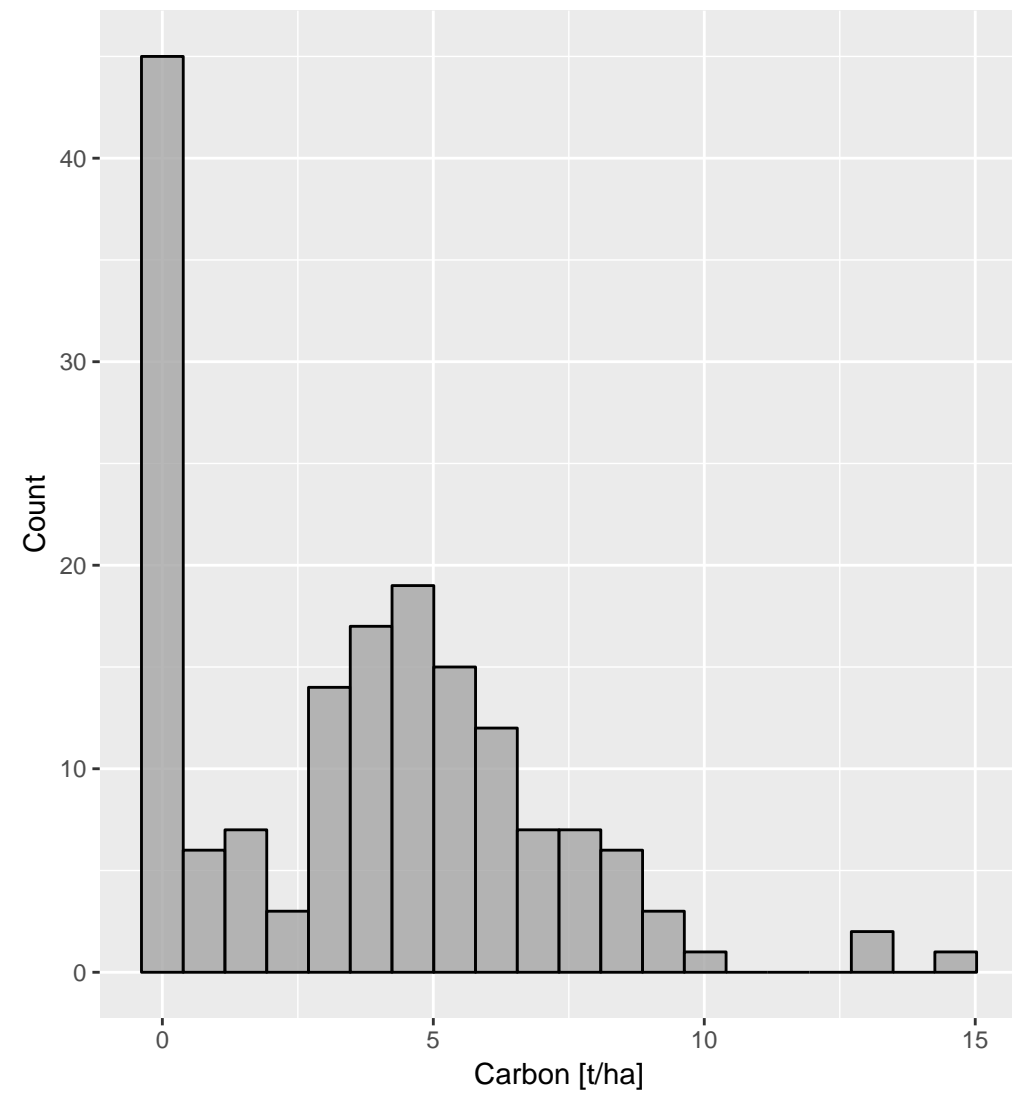

Supplement: Supplementary file 4 — Additional file 4. Histograms of the carbon distribution in the three study sites after the spatial allocation of field samples to the pixel grid. [file 13021_2018_97_MOESM4_ESM.pdf]
